# Supplementary material for: High Drug Resistance Levels Compromise the Control of HIV Infection in Pediatric and Adult Populations in Bata, Equatorial Guinea
Source: Viruses. 2022 Dec 21;15(1):27. doi: 10.3390/v15010027 (PMC9864178; doi:10.3390/v15010027)
Supplement: Supplementary file 1 [file viruses-15-00027-s001.zip › viruses-2024047-supplementary.pdf]

Table S1. Naïve and treated patients with available *pol* sequence.

	No. children/adolescents (%)		No. adults* (%)		Total cohort
**Previous ART-exposure**	No (naïve)	Yes (treated)	No (naïve)	Yes (treated)	
**Number of subjects**	2	56	66	113	237
**Subjects with ≥1 available *pol* HIV-1 sequence**	2 (100)	43 (76.8)	59 (89.4)	37 (32.7)	141 (59.5)
**With PR**	2	38	58	33	131
**With RT**	2	38	51	30	121
**With IN**	2	31	41	23	97
**Only PR**	0	2 (4.7)	5 (8.5)	3 (8.1)	10 (7.1)
**Only RT**	0	1 (2.3)	1 (1.7)	0	2 (1.4)
**Only IN**	0	2 (4.7)	0	2 (5.4)	4 (2.8)
**PR+RT**	0	9 (20.9)	12 (20.3)	11 (29.7)	32 (22.7)
**RT+IN**	0	2 (4.7)	0	2 (5.4)	4 (2.8)
**PR+IN**	0	1 (2.3)	3 (5.1)	2 (5.4)	6 (4.3)
**PR+RT+IN**	2 (100)	26 (60.4)	38 (64.4)	17 (46)	83 (58.9)
**With VL>1000 cp/ml**	2 (100)	43 (100)	59 (100)	37 (100)	141 (100)

Table S1 Legend. ART, antiretroviral therapy; VL, viral load; No, number; PR, protease; RT, reverse transcriptase. *Among the 96 adults with sequence, 7 (7.3%) were pregnant women, 4 treated and 3 naïve at sampling. Analyzed sequences: total cohort (131PR, 121RT and 97IN), naïve cohort (60PR, 53RT, 43IN), treated cohort (71PR, 68RT, 54IN).

Table S2. Naïve and treated patients from Equatorial Guinea with available sequences carrying DRM (2019-2020).

	Available sequences n and sequenced region	With DRM No. (%)	Only major DRM No. (%)	Major+minor DRM No. (%)	Naïve/treated patients with sequences (NCA/TCA/NA/TA)	Naïve children with major DRM No. (%)	Treated children with major DRM No. (%)	Naïve adults with major DRM No. (%)	Treated adults with major DRM No. (%)
**Single resistance**	**141**	**36 (25.5)**	**34 (24.1)**	**36 (25.5)**	**2/43/59/37**	**0**	**7 (16.3)**	**13 (22)**	**14 (37.8)**
**To NRTI**	121 RT	5 (4.1)	6 (5)	5 (4.1)	2/38/51/30	0	1 (2.6)	2 (3.9)	3 (10)
**To NNRTI**	121 RT	25 (20.7)	26 (21.5)	25 (20.7)	2/38/51/30	0	6 (15.8)	10 (19.6)	10 (33.3)
**To PI**	131 PR	2 (1.5)	1 (0.8)	2 (1.5)	2/38/58/33	0	0	0	1 (3)
**To INSTI**	97 IN	3 (3.1)	1 (1)	3 (3.1)	2/31/41/23	0	0	1 (2.4)	0
**Double resistance**	**141**	**27 (19.1)**	**29 (20.6)**	**27 (19.1)**	**2/43/59/37**	**0**	**19 (44.2)**	**0**	**10 (27)**
**NRTI+NNRTI**	121 RT	25 (20.7)	29 (24)	25 (20.7)	2/38/51/30	0	19 (50)	0	10 (33.3)
**NRTI+INSTI**	87 RT+IN	1 (1.1)	0	1 (1.1)	2/28/38/19	0	0	0	0
**NNRTI+INSTI**	87 RT+IN	1 (1.1)	0	1 (1.1)	2/28/38/19	0	0	0	0
**Triple resistance**	**141**	**5 (3.5)**	**1 (0.7)**	**5 (3.5)**	**2/43/59/37**	**0**	**1 (2.3)**	**0**	**0**
**NRTI+NNRT+PI**	115 PR+RT	1 (0.9)	1 (0.9)	1 (0.9)	2/35/50/28	0	1 (2.9)	0	0
**NRTI+NNRTI+INSTI**	87 RT+IN	4 (4.6)	0	4 (4.6)	2/28/38/19	0	0	0	0
**Quadruple resistance**	**141**	**0**	**0**	**0**	**2/43/59/37**	**0**	**0**	**0**	**0**
**NRTI+NNRTI+PI+INSTI**	83	0	0	0	2/26/38/17	0	0	0	0
**Summary resistance by ARV-classes**									
**No DRM**	141	74 (52.5)	77 (54.6)	74 (52.5)	2/43/59/37	2	16 (37.2)	46 (78)	13 (35.1)
**With DRM**	141	67 (47.5)	64 (45.4)	67 (47.5)	2/43/59/37	0	27 (62.8)	13 (22)	24 (64.9)
**NRTI**	121 RT	36 (29.8)	36 (29.8)	36 (29.8)	2/38/51/30	0	21 (55.3)	2 (3.9)	13 (43.3)
**NNRTI**	121 RT	56 (46.3)	56 (46.3)	56 (46.3)	2/38/51/30	0	26 (68.4)	10 (19.6)	20 (66.7)
**PI**	131 PR	3 (2.3)	2 (1.5)	3 (2.3)	2/38/58/33	0	1 (2.6)	0	1 (3)
**INSTI**	97 IN	9 (9.3)	1 (1)	9 (9.3)	2/31/41/23	0	0	1 (2.4)	0

Table S2 legend. Available sequences in 141 participants from Equatorial Guinea under study with DBS samples collected during 2019-2020: 131PR, 121RT and 97IN. No, number; NCA, naïve children and adolescents; TCA, treated children and adolescents; NA, naïve adults; TA, treated adults; only major-DRM, DRM present when only considering major-DRM; major + minor-DRM, DRM present when considering major and minor-DRM; single resistance, to one ARV-class; double resistance, to two ARV-classes; triple resistance, to three ARV-classes; quadruple resistance, to four ARV-classes; no DRM, no DRM found in the available regions per patient. n, number of patients; DRM, drug resistance mutation; ARV, antiretroviral; NRTI, nucleoside reverse transcriptase inhibitors; NNRTI, non-NRTI; PI, protease inhibitors; INSTI, integrase strand transfer inhibitors. We only considered major-DRM to PI, major-DRM to INSTI and DRM to NRTI and to NNRTI, according to Stanford v9.0. Resistance data was absent in all subjects with viraemia below 1000 HIV-1 RNA copies/ml and in 32.2% of those with more than 1000 HIV-1 RNA copies/ml at sampling.

Table S3. DRM in resistant viruses in naïve and treated population from Equatorial Guinea with available *pol* sequence.

DRM	2 naïve children<br>No. (%)	43 naïve<br>No. (%)	59 naïve adults<br>No. (%)	37 treated adults<br>No. (%)
**DRM to NRTI**	**0**<br>**2 RT sequences**	**21 (55.3)**<br>**38 RT sequences**	**2 (3.9)**<br>**51 RT sequences**	**13 (43.3)**<br>**30 RT sequences**
**M184V/I**	0	19/1 (52.6)	0	10/0 (33.3)
**K219Q/N/E/R**	0	0/1/2/2 (13.2)	0	1/1/3/1 (20)
**T215F/Y/P/SY**	0	4/2/1/1 (21.1)	0	1/0/0/0 (3.3)
**K70R/E**	0	3/0 (7.9)	0	3/1 (13.3)
**M41L**	0	5 (13.2)	1 (2)	2 (6.7)
**A62V, K65R**	0	1 (2.6)	0	2 (6.7)
**D67N/S/G**	0	0/1/1 (5.3)	0	1/0/0 (3.3)
**E44D**	0	2 (5.3)	1 (2)	0
**L74I**	0	2 (5.3)	0	0
**F77L, L210W**	0	1 (2.6)	0	0
**DRM to NNRTI**	**0**<br>**2 RT sequences**	**26 (68.4)**<br>**38 RT sequences**	**10 (19.6)**<br>**51 RT sequences**	**20 (66.7)**<br>**30 RT sequences**
**K103N/H/S**	0	14/0/2 (42.1)	4/0/0 (7.8)	15/1/0 (53.3)
**G190S/A**	0	0/9 (23.7)	0/1 (2)	1/1 (6.7)
**A98G**	0	6 (15.8)	0	5 (16.7)
**V108I**	0	8 (21.1)	0	3 (10)
**Y181C**	0	5 (13.2)	0	3 (10)
**P225H**	0	2 (5.3)	0	6 (20)
**K101H/E**	0	1/2 (7.9)	0/1 (2)	1/1 (6.7)
**F227L**	0	4 (10.5)	0	0
**H221Y**	0	2 (5.3)	0	1 (3.3)
**K238T/N**	0	0/1 (2.6)	0	2/0 (6.7)
**V179E/D**	0	1/0 (2.6)	5/1 (11.8)	1/0 (3.3)
**V106I**	0	1 (2.6)	1 (2)	1 (3.3)
**Y318F, Y188L**	0	2 (5.3)	0	0
**M230L/I**	0	0/1 (2.6)	0	1/0 (3.3)
**E138A/Q**	0	0/1 (2.6)	2/0 (3.9)	0
**L234I**	0	0	0	1 (3.3)
**DRM to PI**	**0**<br>2 PR sequences	**1 (2.6)**<br>38 PR sequences	**1 (1.7)**<br>58 PR sequences	**1 (3)**<br>33 PR sequences
**Major**	0	1 (2.6)	0	1 (3)
**M46L**	0	1 (2.6)	0	1 (3)
**I54V, L76V, I84V**	0	1 (2.6)	0	0
**Minor**	0	0	1 (1.7)	0
**L89V**	0	0	1 (1.7)	0
**DRM to INSTI**	**0**<br>2 IN sequences	**1 (3.2)**<br>31 IN sequences	**4 (9.8)**<br>41 IN sequences	**4 (17.4)**<br>23 IN sequences
**Major**	0	0	1 (2.4)	0
**E92K**	0	0	1 (2.4)	0
**Minor**	0	1 (3.2)	3 (7.3)	4 (17.4)
**T97A**	0	1 (3.2)	0	3 (13)
**E157Q**	0	0	2 (4.9)	1 (4.3)
**G163K**	0	0	1 (2.4)	0

Table S3 Legend. Available sequences in 141 subjects under study: 131PR, 121RT and 96IN. No, number; DRM, drug resistance mutation; NRTI, nucleoside reverse transcriptase inhibitors; NNRTI, non-NRTI; PI, protease

inhibitors; INSTI, integrase strand transfer inhibitors. We considered major and minor-DRM to PI, INSTI, NRTI and NNRTI, according to Stanford v9.0. DRM to INSTI E92K is an APOBEC mutation identified as a major-DRM by Stanford.

Table S4. Comparison of studies reporting HIV-1 resistance in Equatorial Guinea.

	Djoko *et al.* 2010 [30] No. (%)	Yebra *et al.*[#] 2013 [32] No. (%)	Alvarez *et al.* 2016 [29] No. (%)	This study No. (%)	P value
**Journal PMID**	20718620	23717585	27798676		
**Reference**	30	32	29		
**Sampling year**	2008	1997-2011	2012-2013	2019-2020	
**Population from EG**	Military	Equatoguineans immigrants living in Spain	Pregnant women	General	
**% adults with sequence**	41 (85.4)	186 (95.4)	38 (100)	96 (68.1)	
**% children-adolescents with sequence**	0	9 (4.6)	0	45 (31.9)	
**Number of naïve/treated patients with *pol* sequence**	41/0	148/31	8/30	61/80	
**TDR in naïve***	2 (4.8)	7 (4.7)	0	6 (9.8)	ns
**To NRTI**	1 (2.4)	2 (1.8)	0	1 (1.9)	ns
**To NNRTI**	0	1 (0.9)	0	5 (9.4)	*
**To PI**	1 (2.4)	5 (3.4)	0	0	ns
**To INSTI**	-	-	-	0	
**DRM in treated**	-	9 (29)	6 (20)	51 (63.8)	***
**To NRTI**	-	3 (16.7)	2 (8.7)	34 (50)	**
**To NNRTI**	-	5 (27.8)	3 (13)	46 (67.6)	***
**To PI**	-	5 (16.1)	1 (3.7)	2 (2.8)	*
**To INSTI**	-	-	-	0	

Table S4 Legend. No, number; EG, Equatorial Guinea; naïve, ART-naïve, treated, ART-treated; *TDR, transmitted resistance mutations by Calibrated Population Resistance tool at Stanford HIV website; DRM, drug resistance mutations by Stanford algorithm; NRTI, nucleoside reverse transcriptase inhibitors; NNRTI, non-NRTI; PI, protease inhibitors; INSTI, integrase strand transfer inhibitors; dash, not done. # In Yebra et al [32], the percentages were calculated according to the experience to each ARV-class, while in this table we considered as naïve subjects these with no previous experience to any ARV-family at sampling, and treated these with experience to at least one ARV-class; ns, not significant (P>0.05); significant P values: *, < 0.05; **, < 0.001; ***, <0.0001.

Table S5. Comparison of studies reporting HIV-1 variants in Equatorial Guinea.

	Yebra et al. 2013 [32] No. (%)	Alvarez et al. 2016 [29] No. (%)	This study No. (%)	P value
**Journal PMID**	23717585	27798676		
**Sampling year**	1997-2011	2012-2013	2019-2020	
**Number of patients**	278	69	237	
**% adults**	100%	100%	75.5%	
**Patients with *pol* sequence**	278 (100)	38 (55.1)	141 (59.5)	
**HIV-1 variants**				
**Pure subtypes**	122 (43.9)	9 (23.7)	20 (14.1)	***
**A**	38 (13.7)	1 A3 (2.6)	1 A1/5 A3 (4.3)	*
**B**	20 (7.2)	1 (2.6)	1 (0.7)	*
**C**	16 (5.7)	4 (10.5)	5 (3.5)	ns
**D**	14 (5)	1 (2.6)	1 (0.7)	ns
**F**	11 (4)	1 F2 (2.6)	1 F2 (0.7)	ns
**G**	15 (5.4)	1 (2.6)	4 (2.8)	ns
**H**	8 (2.9)	0	1 (0.7)	ns
**K**	0	0	1 (0.7)	
**Recombinants**	156 (56.1)	29 (76.3)	121 (85.8)	***
**CRF02_AG**	133 (47.8)	21 (55.2)	76 (53.9)	ns
**CRF06_cpx**	4 (1.4)	1 (2.6)	3 (2.2)	ns
**CRF09_cpx**	1 (0.4)	0	0	ns
**CRF11_cpx**	7 (2.5)	1 (2.6)	0	ns
**CRF13_cpx**	3 (1.1)	0	1 (0.7)	ns
**CRF18_cpx**	1 (0.4)	0	0	ns
**CRF22_01A1**	3 (1.1)	4 (10.5)	4 (2.8)	*
**CRF26_AU**	0	0	5 (3.5)	*
**CRF37_cpx**	0	0	2 (1.5)	ns
**CPR45_cpx**	0	0	2 (1.5)	ns
**URF**	4 (1.4)	2 (5.3)	28 (19.9)	***

Table S5 Legend. No, number; %, percentage; CRF, circulating recombinant forms; URF, unique recombinant forms; ns, not significant (P>0.05); *, < 0.05; ***, <0.0001.

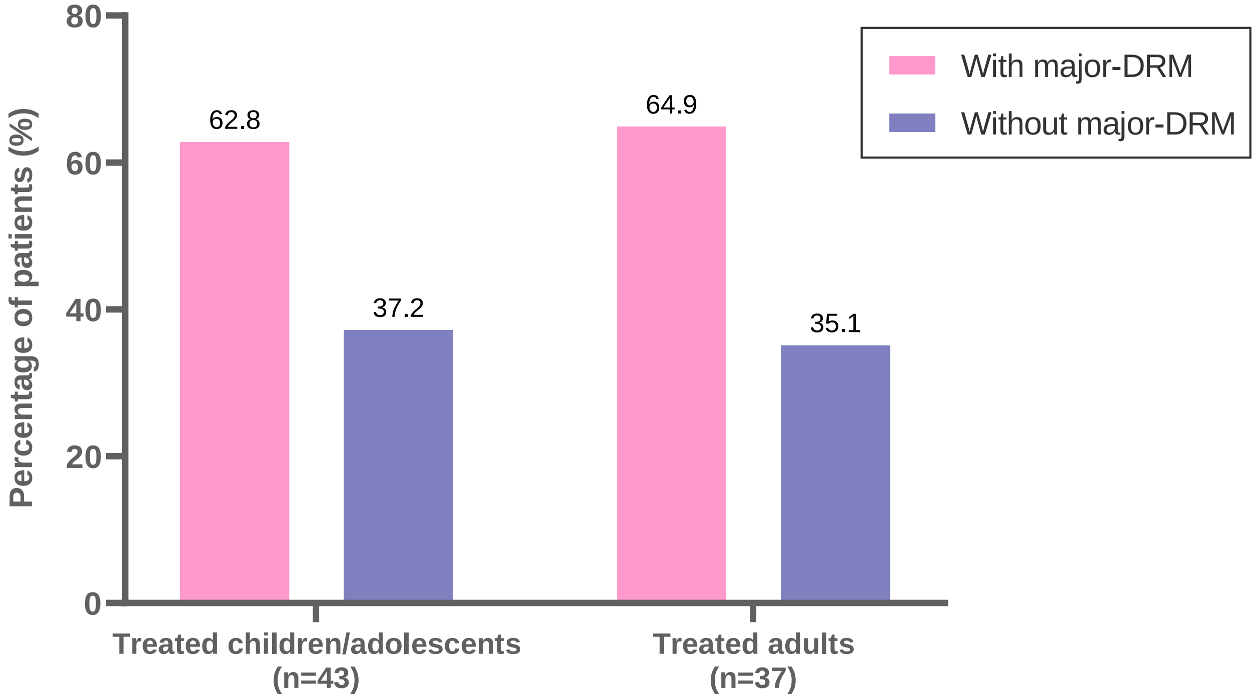

Figure S1. Percentage of ART-treated patients under therapeutic failure with *vs*. without DRM among the 80 treated patients under study. Available sequences in 43 children/adolescents and 37 adults. ART, antiretroviral; treated, ART-treated; DRM, drug resistance mutations.

ID	Protease Inhibitors (PI)								Nucleoside Reverse Transcriptase Inhibitors (NRTI)							Non-nucleoside Reverse Transcriptase Inhibitors (NNRTI)					Integrase Strand Transfer Inhibitors (INSTI)				
	ATV/r	DRV/r	FPV/r	IDV/r	LPV/r	NFV	SQV/r	TPV/r	ABC	AZT	D4T	DDI	FTC	3TC	TDF	DOR	EFV	ETR	NVP	RPV	BIC	CAB	DTG	EVG	RAL
NAÏVE CHILDREN																									
19.GE.NN.01																									
19.GE.NN.21																									
TREATED CHILDREN																									
19.GE.NT.01					x					x				x			x								
19.GE.NT.02										x				x			x								
19.GE.NT.03										x			x	x	x		x		x						
19.GE.NT.04										x				x			x								
19.GE.NT.05														x	x								x		
19.GE.NT.07										x				x					x						
19.GE.NT.09										x				x					x						
19.GE.NT.10										x				x			x		x						
19.GE.NT.11					x					x	x			x			x		x						
19.GE.NT.12										x				x			x								
19.GE.NT.13										x				x			x		x						

ID	Protease Inhibitors (PI)								Nucleoside Reverse Transcriptase Inhibitors (NRTI)							Non-nucleoside Reverse Transcriptase Inhibitors (NNRTI)					Integrase Strand Transfer Inhibitors (INSTI)				
	ATV/r	DRV/r	FPV/r	IDV/r	LPV/r	NFV	SQV/r	TPV/r	ABC	AZT	D4T	DDI	FTC	3TC	TDF	DOR	EFV	ETR	NVP	RPV	BIC	CAB	DTG	EVG	RAL
19.GE.NT.14					x					x				x	x		x		x						
19.GE.NT.15					x					x				x	x										
19.GE.NT.16										x				x					x						
19.GE.NT.17									x	x				x			x		x						
19.GE.NT.18										x				x			x								
19.GE.NT.19										x				x					x						
19.GE.NT.20										x				x					x						
19.GE.NT.23											x		x	x	x		x		x						
19.GE.NT.24										x	x			x			x		x						
19.GE.NT.25										x			x	x	x		x								
19.GE.NT.26										x				x	x		x		x						
19.GE.NT.27																									
19.GE.NT.28					x				x	x				x	x				x						
19.GE.NT.29										x				x			x								
19.GE.NT.30										x				x			x		x						
19.GE.NT.31																									

ID	Protease Inhibitors (PI)								Nucleoside Reverse Transcriptase Inhibitors (NRTI)							Non-nucleoside Reverse Transcriptase Inhibitors (NNRTI)					Integrase Strand Transfer Inhibitors (INSTI)				
	ATV/r	DRV/r	FPV/r	IDV/r	LPV/r	NFV	SQV/r	TPV/r	ABC	AZT	D4T	DDI	FTC	3TC	TDF	DOR	EFV	ETR	NVP	RPV	BIC	CAB	DTG	EVG	RAL
19.GE.NT.32										x				x					x						
19.GE.NT.34																									
19.GE.NT.37																									
19.GE.NT.39																									
19.GE.NT.40														x	x		x								
19.GE.NT.41										x				x			x								
19.GE.NT.42										x				x			x								
19.GE.NT.43					x						x		x	x	x		x								
19.GE.NT.44																									
19.GE.NT.45										x				x			x		x						
19.GE.NT.48					x					x				x	x		x								
19.GE.NT.49																									
19.GE.NT.50										x				x					x						
19.GE.NT.55									x	x				x			x		x						
19.GE.NT.59										x				x	x				x				x		
19.GE.NT.60										x				x			x		x						

ID	Protease Inhibitors (PI)								Nucleoside Reverse Transcriptase Inhibitors (NRTI)							Non-nucleoside Reverse Transcriptase Inhibitors (NNRTI)					Integrase Strand Transfer Inhibitors (INSTI)				
	ATV/r	DRV/r	FPV/r	IDV/r	LPV/r	NFV	SQV/r	TPV/r	ABC	AZT	D4T	DDI	FTC	3TC	TDF	DOR	EFV	ETR	NVP	RPV	BIC	CAB	DTG	EVG	RAL
NAÏVE ADULTS																									
19.GE.AN.02																									
19.GE.AN.03																									
19.GE.AN.05																									
19.GE.AN.06																									
19.GE.AN.07																									
19.GE.AN.10																									
19.GE.AN.11																									
19.GE.AN.14																									
19.GE.AN.15																									
19.GE.AN.16																									
19.GE.AN.17																									
19.GE.AN.18																									
19.GE.AN.21																									
19.GE.AN.22																									
19.GE.AN.23																									

ID	Protease Inhibitors (PI)								Nucleoside Reverse Transcriptase Inhibitors (NRTI)							Non-nucleoside Reverse Transcriptase Inhibitors (NNRTI)					Integrase Strand Transfer Inhibitors (INSTI)				
	ATV/r	DRV/r	FPV/r	IDV/r	LPV/r	NFV	SQV/r	TPV/r	ABC	AZT	D4T	DDI	FTC	3TC	TDF	DOR	EFV	ETR	NVP	RPV	BIC	CAB	DTG	EVG	RAL
19.GE.AN.24																									
19.GE.AN.25																									
19.GE.AN.27																									
19.GE.AN.28																									
19.GE.AN.38																									
19.GE.AN.39																									
19.GE.AN.40																									
19.GE.AN.41																									
19.GE.AN.42																									
19.GE.AN.43																									
19.GE.AN.44																									
19.GE.AN.47																									
19.GE.AN.48																									
19.GE.AN.49																									
19.GE.AN.50																									
19.GE.AN.51																									

ID	Protease Inhibitors (PI)								Nucleoside Reverse Transcriptase Inhibitors (NRTI)							Non-nucleoside Reverse Transcriptase Inhibitors (NNRTI)					Integrase Strand Transfer Inhibitors (INSTI)				
	ATV/r	DRV/r	FPV/r	IDV/r	LPV/r	NFV	SQV/r	TPV/r	ABC	AZT	D4T	DDI	FTC	3TC	TDF	DOR	EFV	ETR	NVP	RPV	BIC	CAB	DTG	EVG	RAL
19.GE.AN.52																									
19.GE.AN.53																									
19.GE.AN.54																									
19.GE.AN.55																									
19.GE.AN.57																									
19.GE.AN.58																									
19.GE.AN.59																									
19.GE.AN.60																									
19.GE.AN.61																									
19.GE.AN.63																									
19.GE.AN.64																									
19.GE.AN.65																									
19.GE.AN.67																									
19.GE.AN.68																									
19.GE.AN.69																									
19.GE.AN.70																									

ID	Protease Inhibitors (PI)								Nucleoside Reverse Transcriptase Inhibitors (NRTI)							Non-nucleoside Reverse Transcriptase Inhibitors (NNRTI)					Integrase Strand Transfer Inhibitors (INSTI)				
	ATV/r	DRV/r	FPV/r	IDV/r	LPV/r	NFV	SQV/r	TPV/r	ABC	AZT	D4T	DDI	FTC	3TC	TDF	DOR	EFV	ETR	NVP	RPV	BIC	CAB	DTG	EVG	RAL
19.GE.AN.71																									
19.GE.AN.73																									
19.GE.AN.74																									
19.GE.AN.75																									
19.GE.AN.76																									
19.GE.AN.77																									
19.GE.AN.78																									
19.GE.AN.79																									
19.GE.AN.80																									
19.GE.EN.02																									
19.GE.EN.05																									
19.GE.EN.07																									
TREATED ADULTS																									
19.GE.AT.12					x					x			x	x	x		x								
19.GE.AT.13													x		x		x								
19.GE.AT.20													x	x	x		x								

ID	Protease Inhibitors (PI)								Nucleoside Reverse Transcriptase Inhibitors (NRTI)							Non-nucleoside Reverse Transcriptase Inhibitors (NNRTI)					Integrase Strand Transfer Inhibitors (INSTI)				
	ATV/r	DRV/r	FPV/r	IDV/r	LPV/r	NFV	SQV/r	TPV/r	ABC	AZT	D4T	DDI	FTC	3TC	TDF	DOR	EFV	ETR	NVP	RPV	BIC	CAB	DTG	EVG	RAL
19.GE.AT.22					x						x			x	x		x		x						
19.GE.AT.24					x					x	x			x	x		x		x				x		
19.GE.AT.29											x		x	x	x		x		x						
19.GE.AT.30													x	x	x		x						x		
19.GE.AT.32														x	x		x								
19.GE.AT.33										x			x	x	x				x				x		
19.GE.AT.34					x					x	x		x	x	x		x		x						
19.GE.AT.38																									
19.GE.AT.39											x		x	x	x		x		x				x		
19.GE.AT.40										x	x			x					x						
19.GE.AT.46					x						x		x	x	x		x		x						
19.GE.AT.47														x	x		x								
19.GE.AT.51					x					x				x	x				x						
19.GE.AT.52													x	x	x		x						x		
19.GE.AT.55										x	x			x	x		x		x						
19.GE.AT.57														x	x		x								

ID	Protease Inhibitors (PI)								Nucleoside Reverse Transcriptase Inhibitors (NRTI)							Non-nucleoside Reverse Transcriptase Inhibitors (NNRTI)					Integrase Strand Transfer Inhibitors (INSTI)				
	ATV/r	DRV/r	FPV/r	IDV/r	LPV/r	NFV	SQV/r	TPV/r	ABC	AZT	D4T	DDI	FTC	3TC	TDF	DOR	EFV	ETR	NVP	RPV	BIC	CAB	DTG	EVG	RAL
19.GE.ET.05														X	X		X								
19.GE.ET.07										X	X			X					X						
19.GE.ET.13																									
19.GE.ET.14																									
19.GE.MT.11																									
19.GE.MT.12																									
19.GE.MT.13																									
19.GE.MT.16																									
19.GE.MT.24																									
19.GE.MT.25																									
19.GE.MT.31																									
19.GE.MT.35																									
19.GE.MT.39																									
19.GE.MT.40																									
19.GE.MT.41																									
19.GE.MT.42																									

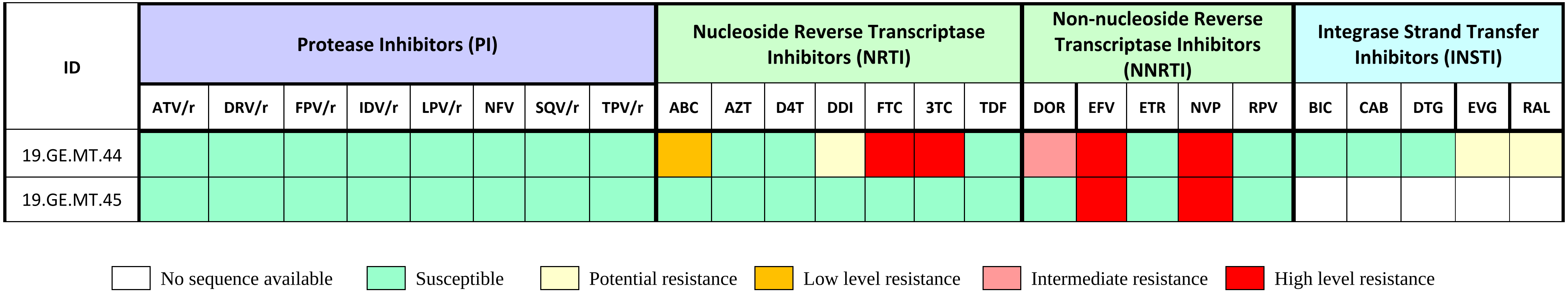

Figure S2. ARV susceptibility by Stanford in 141 patients samples with available *pol* sequence. Predicted ARV susceptibility in 141 available sequences (131PR/121RT/97IN) and antiretroviral experience in each patient from Equatorial Guinea with available sequence under study. Patients' ID codes were provided in the laboratory after sample reception to maintain their anonymity. x, antiretroviral experience; NRTI, nucleoside reverse transcriptase inhibitors; NNRTI, non-NRTI; PI, protease inhibitors; INSTI, integrase strand transfer inhibitors. ATV/r, atazanavir/ritonavir; DRV/r, darunavir/ritonavir; FPV/r, fosamprenavir/ritonavir; IDV/r, indinavir/ritonavir; LPV/r, lopinavir/ritonavir; NFV, nelfinavir; SQV/r, saquinavir/ritonavir; TPV/r, tipranavir/ritonavir; ABC, abacavir; AZT, zidovudine; D4T, stavudine; DDI, didanosine; FTC, emtricitabine; 3TC, lamivudine; TDF, tenofovir; DOR, doravirine; EFV, efavirenz; ETR, etravirine; NVP, nevirapine; RPV, rilpivirine; BIC, bictegravir; CAB, cabotegravir; DTG, dolutegravir; EVG, elvitegravir; RAL, raltegravir.
